# Chondrosarcoma With Pulmonary Metastatic Calcifications: A Case Report and Review of the Literature

**DOI:** 10.7759/cureus.53234

**Published:** 2024-01-30

**Authors:** Paul F Hanona, Daniel Ezekwudo, Joseph Anderson, Ishmael Jaiyesimi

**Affiliations:** 1 Hematology and Oncology, Corewell Health William Beaumont University Hospital, Royal Oak, USA

**Keywords:** cartilage [c26.411], bone cancer, rare cancers, metastatic calcifications, high-grade chondrosarcoma

## Abstract

A chondrosarcoma with pulmonary metastatic calcifications is a rarely reported phenomenon. This report discusses chondrosarcomas and their clinical features, diagnosis, and treatment, using as an example the case of a 55-year-old female with a right pelvic chondrosarcoma that developed over 10 years. In the last two years, the patient had increasing pulmonary findings, including pulmonary nodules, ground glass opacities, and likely pulmonary metastatic calcifications. The objective of this report is to explore chondrosarcomas and their pattern of metastatic presentation, with the hope of improving recognition of the disease and streamlining treatment.

## Introduction

Chondrosarcomas are defined as a group of bone cancers that develop in cartilage cells. A quarter of all osseous neoplasms are chondrosarcomas. Typically, chondrosarcomas are asymptomatic [1]. However, chondrosarcomas can limit function, cause pain, and result in deformity as well as increase the risk of fracture. Histological grading largely reflects the behavior and overall survival associated with chondrosarcomas. Briefly, chondrosarcomas are graded on a scale of 1-3, with a higher grade indicating higher cellularity, higher malignant potential, and worse overall prognosis [2,3]. Various types of chondrosarcomas are distributed among the pelvis, femur, humerus, ribs, shoulders, jaw bones, and vertebra [4]. 

Chondrosarcomas are typically detected using conventional radiographs, magnetic resonance imaging (MRI), computed tomography (CT) scans, and diagnostic biopsies [5-7]. The most common treatment is surgical resection, with the goal of maintaining as much function as possible [8]. After surgical resection, some chondrosarcomas may require radiotherapy treatment [9]. Likewise, the utility of chemotherapy depends on the type of chondrosarcoma, with it being used both in the neoadjuvant and adjuvant settings. Patients with advanced unresectable or metastatic disease can benefit from palliation or clinical trials depending on the case [10]. 

Calcifications in the lung occur from a variety of etiologies. They most often occur in the setting of end-stage renal disease, or otherwise, during infection, amyloidosis, sarcoidosis, or other fibrotic lung diseases [11]. There have been reported cases of malignancy associated with hypercalcemia, which leads to calcification of the lung [12]. The association between chondrosarcomas and pulmonary metastatic calcification is a phenomenon that is rarely reported when compared to other causes of calcification in the lung. 

## Case presentation

A 55-year-old female with a history of chondrosarcoma status post right hemipelvectomy in 2013, chronic kidney disease, recurrent deep venous thrombosis, and warfarin-induced skin necrosis presented with urinary bleeding. In July 2022 two days prior to presentation, a percutaneous nephroureteral tube and a bladder catheter were removed, with increased urinary bleeding noted thereafter. Of note, her hemipelvectomy in 2013 involved bladder injury, distal ureterectomy, bladder repair, ureteral diversion, abdominal compartment syndrome, ileum perforation, and small bowel resection with ileostomy and colostomy, leading to the requirement for chronic tubing in her urinary tract. Related to her current urinary hematuria, her CT scan showed a small amount of perinephric/subcapsular hemorrhage along the anterior inferior aspect of the left kidney. CT also showed a recurrent soft tissue nodularity along the right aspect of the pelvis in the location of the previous hemipelvectomy, presumably related to recurrent/metastatic chondrosarcoma. 

**Figure 1:**
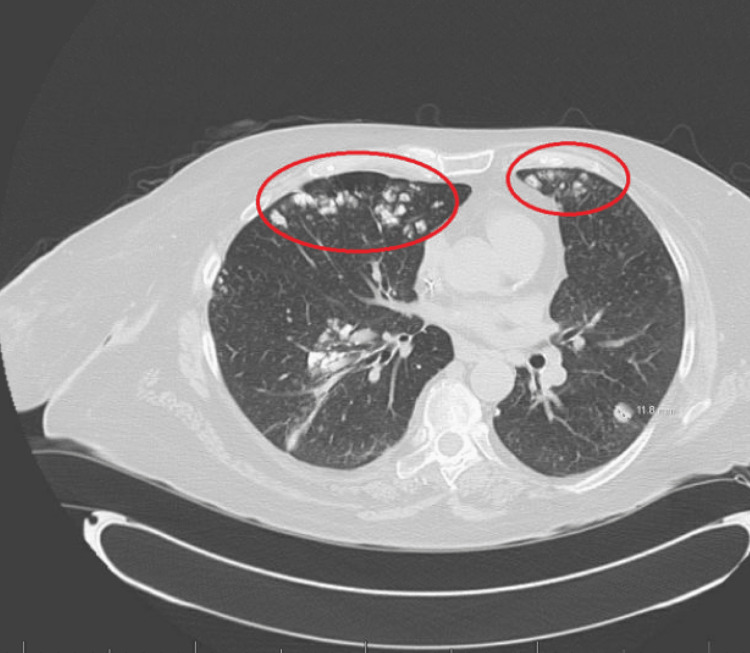
CT chest scan with contrast in October of 2020, showing bilateral anterior and apical pulmonary nodules and ground glass opacities.

**Figure 2:**
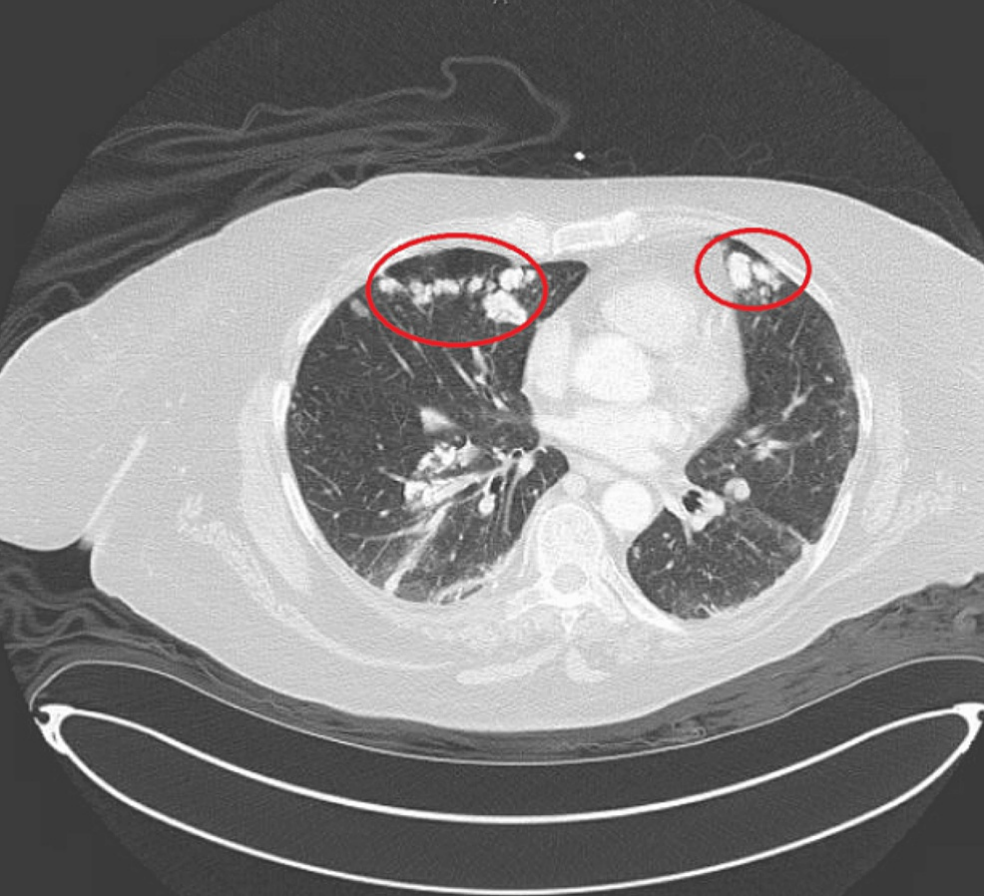
CT chest scan from April 2022 considered the potential for pulmonary metastatic calcifications situated in the anterior part of the left and the right lung (red circles), potentially from a recurrent right pelvic chondrosarcoma. Importantly, the size of the nodules has increased from the previous CT scan.

**Figure 3:**
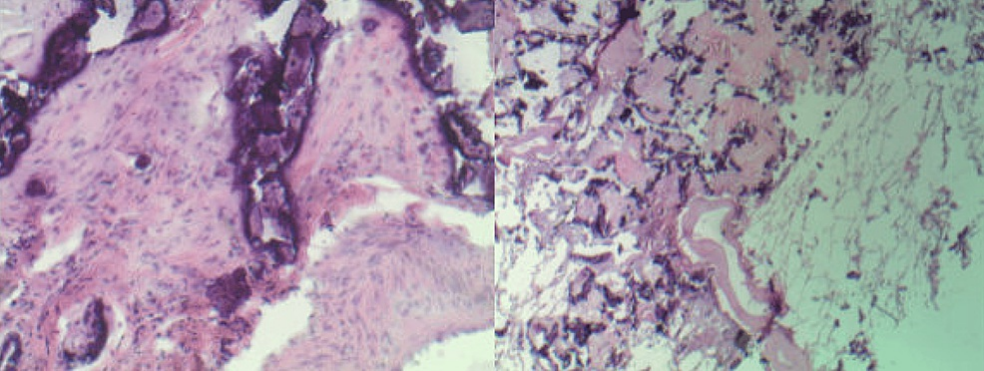
Lung wedge biopsy of the left upper lobe was performed. Sectioning reveals a 5.5 x 2.7 x 1.5 cm calcified gray-white, rubbery mass/nodule, lesion. Microscopic examination of the lesion reviewed extensive calcification and fibrosis as shown in slide one (stained with H&E, at 40 x magnification) and slide 2 (stained with H&E, at 200 x magnification). The calcified lesion represents calcified pulmonary metastases of chondrosarcoma.

The 2013 initial presentation of chondrosarcoma included right pelvic pain, with a CT scan showing a 5 cm right pelvic mass involving the symphysis, which resulted in surgical resection with pathology that revealed a chondrosarcoma grade 2. Since the original resection, the patient has had six recurrences of the chondrosarcoma with six resections in the same right pelvic region. The patient also received radiotherapy to the right pelvis in 2019 (six years after the initial presentation). Her first CT of the chest in October 2020 showed no findings concerning metastases (Figure 1). However, concurrently a skin biopsy showed calcification of small vessels and interstitial calcifications. A CT scan in February 2021 showed a few ground glass opacities bilaterally. A CT scan in August 2021 showed increasing bilateral pulmonary nodules. A lung nodule biopsy performed at that time showed patchy calcifications with a giant cell reaction indicating inflammatory nonspecific findings; however, no malignancy was identified. Another lung nodule biopsy performed in July 2021 showed multiple dystrophic microcalcifications of the blood vessel but no neoplasia. A CT scan done in April 2022 started to suggest pulmonary metastatic calcifications (Figure 2). A left upper lobe wedge resection performed in July 2022 showed extensive calcification and fibrosis with negative immunostaining for AE1/3, CD34, SMA, desmin, ERG, STAT6, and S100 (Figure 3). Those findings likely represented pulmonary metastases of chondrosarcoma, or less likely, pulmonary dystrophic calcification. The patient then was treated by a nephrologist for cutaneous calciphylaxis with sodium thiosulfate, with improvement. This further suggested that the pathology reflected calcified pulmonary metastases of a chondrosarcoma. 

**Figure 4:**
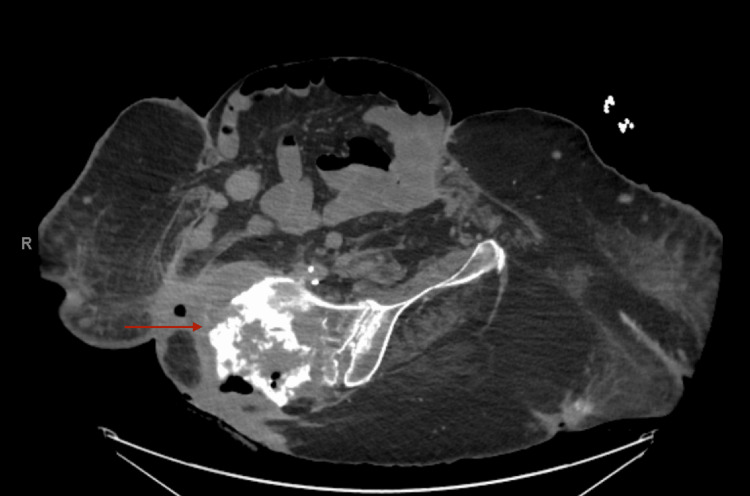
The red arrow points to the area of the pelvis most concerning chondrosarcoma growth and destruction. Of note, the patient was rotated in this picture.

The physical exam upon presentation in July 2022 was largely unremarkable, apart from hematuria in the newly placed left and right percutaneous nephroureteral tubes. The right lower extremity and the left upper extremity from the elbow down were surgically absent. A wound in the right hip was thought to be a source of infection. Pertinent laboratory values included a hemoglobin of 8.6 g/dL, slightly elevated aspartate transaminase (AST) of 85 IU/L, and alanine transaminase (ALT) of 76 IU/L; otherwise, blood chemistry was largely stable. The patient over the course of her admission did not require blood transfusions. Her blood culture grew coagulase-negative Staphylococcus, and antibiotic treatment was initiated. No chest CT was performed during that admission due to a lack of active thoracic complaints. An oncology consultation was performed to evaluate the possible recurrence of the chondrosarcoma as well as the potential for calcified pulmonary metastases in the nodules seen on the CT three months prior to admission. A repeat lung biopsy was not felt to be necessary, as there was already suspicion of pulmonary metastatic calcifications. The patient was discharged after treatment for her right hip wound causing sepsis and hematuria. She began treatment with the kinase inhibitor pazopanib in the outpatient setting. She returned to the hospital one month later with sepsis, and a CT at that time showed an increasing pelvic mass concerning chondrosarcoma growth, and new liver and uterine mass lesions (Figure 4). Biopsy of the liver mass revealed adenocarcinoma likely metastasized from primary lung cancer since the tissue was TTF-1 positive. This finding likely indicated a double primary. Biopsy of the uterine mass showed scant fragments of mesenchymal tissue. Ultimately, the patient’s sepsis progressed, with worsening kidney damage and ultimate septic shock. She was transitioned to hospice and passed away shortly after.

## Discussion

This is the report of a 55-year-old female with an interesting case of right-sided pelvic chondrosarcoma with likely pulmonary metastatic calcifications to the bilateral lungs. Most chondrosarcomas are low-grade and have low metastatic potential. The higher the grade of the original chondrosarcoma, the more likely it is to metastasize to the lungs. Some reports show that grade 3 chondrosarcomas have more than a 50% chance of metastasizing to the lung [13,14]. Our patient had grade 2 chondrosarcoma, yet still had metastasis to the lungs. In addition, individuals with higher-grade chondrosarcoma typically have a quicker timeline to lung metastasis than those with low-grade disease. Some features that portend a likely diagnosis of pulmonary metastatic calcifications include nodules greater than 10 mm in diameter, irregular calcification, and bilateral nodules. The location of the nodules can be either peripheral or central [15]. The patient described had bilateral nodules up to 18mm in diameter. 

A previous report of a 64-year-old man with a primarily palatal chondrosarcoma that metastasized to the lung described a nodule of about 35 mm in diameter with eccentric calcifications in the left upper lobe of the lung. The eccentric nature of the calcification simplified treatment planning and surgical resection because it was more apparent that the nodule was likely a metastatic calcification [16]. In contrast, our patient had calcifications in the lung that were not obviously eccentric. This made a confident radiological diagnosis difficult. Our patient also had end-stage renal disease, which made consideration of calciphylaxis to the lung a strong alternative to a diagnosis of metastatic calcifications in the lung. 

The timeline of the lung metastasis may also increase the difficulty of diagnosis, with some reports showing that the chondrosarcoma resected from rib bones can later present as metastatic pulmonary nodules [17]. Our case involved a patient who had undergone several pelvic chondrosarcoma resections before any pulmonary findings were noted on chest CT scans. Another consideration in the era of the coronavirus disease 2019 (COVID-19) pandemic is being able to accurately differentiate between ground glass opacities from COVID-19 and pulmonary metastatic calcifications from chondrosarcomas. One published report described a patient who had both COVID-19 and a primary chondrosarcoma that had spread to the lungs. After no improvement resulted from COVID-19-directed treatment, a biopsy of the lung nodule proved pulmonary metastases of a chondrosarcoma origin [18]. It is unknown whether our patient ever had COVID-19, but it could have potentially clouded the interpretation of the 2021 CT chest that did not consider pulmonary metastatic calcifications as part of the differential diagnosis. 

Treatment of chondrosarcomas mainly involves surgical resection [19]. Often, chondrosarcomas can be rapidly growing and surgical delay can lead to significant morbidity. One publication reported a case of a 45-year-old with a primarily right humeral chondrosarcoma that had been surgically removed multiple times, exhibited multiple recurrences with metastasis to the lungs, and eventually spread to the cardiac tissue. This caused an acute heart failure episode that required emergent resection of the pulmonary metastases [20]. Usually, lung metastasectomy is reserved for those with grade 2 or 3 chondrosarcomas that have spread; however, when compared to chemotherapy and radiotherapy, better patient outcomes can be achieved with pulmonary metastasectomy. One study showed that the five-year survival of those who had undergone pulmonary metastasectomy was 55%, which was superior to the 13% five-year survival for those who had undergone combination chemoradiotherapy [21]. As our understanding of molecular therapy advances, therapies targeting IDH1/2 and COL2A1 have been linked to successful treatment of chondrosarcomas [22,23]. Immunotherapies such as dasatinib have proved to be helpful in treatment, although research remains ongoing [24]. Our patient received pazopanib, a kinase inhibitor targeting VEGFR, platelet-derived growth factor receptor, and c-kit, which recent research shows also can be helpful in advanced chondrosarcomas, although our patient had metastatic pulmonary calcifications which are not commonly included in clinical trials [25]. 

## Conclusions

Our patient received treatment for her right pelvic chondrosarcoma over ten years. In the last two years, she had increasing pulmonary findings consistent with metastatic calcifications of the lungs. Radiological diagnosis via CT can be a challenge because there are no set criteria. The literature on metastatic calcification of the lungs has been largely limited to case reports, which together exemplify a pattern of presentation similar to our patient’s case. Our hope in sharing this case is to add to the literature already available to make future recognition of this disease more straightforward. 
